# TRIM21 Polymorphisms are associated with Susceptibility and Clinical Status of Oral Squamous Cell Carcinoma patients

**DOI:** 10.7150/ijms.56614

**Published:** 2021-06-11

**Authors:** Chun-Yi Chuang, Yi-Chung Chien, Chiao-Wen Lin, Chia-Hsuan Chou, Shuo-Chueh Chen, Chun-Lin Liu, Li-Yuan Bai, Shun-Fa Yang, Yung-Luen Yu

**Affiliations:** 1 School of Medicine, Chung Shan Medical University, Taichung 40201, Taiwan.; 2 Department of Otolaryngology, Chung Shan Medical University Hospital, Taichung 40201, Taiwan.; 3 Graduate Institute of Biomedical Sciences, China Medical University, Taichung 40402, Taiwan.; 4 Ph.D. Program for Translational Medicine, China Medical University, Taichung 40402, Taiwan.; 5 Institute of New Drug Development, China Medical University, Taichung, 404, Taiwan.; 6 Drug Development Center, Research Center for Cancer Biology, China Medical University, Taichung, 404, Taiwan.; 7 Center for Molecular Medicine, China Medical University Hospital, Taichung 40402, Taiwan.; 8 Institute of Oral Sciences, Chung Shan Medical University, Taichung 40201, Taiwan.; 9 Department of Dentistry, Chung Shan Medical University Hospital 40201, Taichung, Taiwan.; 10 Institute of Medicine, Chung Shan Medical University, Taichung 40201, Taiwan.; 11 Department of Medical Research, Chung Shan Medical University Hospital, Taichung 40201, Taiwan.; 12 Division of Chest Medicine, Department of Internal Medicine, China Medical University Hospital, Taichung 40402, Taiwan.; 13 Department of Neurosurgery, China Medical University Hospital, Taichung 40402, Taiwan.; 14 Division of Hematology and Oncology, Department of Internal Medicine, China Medical University Hospital, Taichung 40402, Taiwan.; 15 Department of Medical Laboratory Science and Biotechnology, Asia University, Taichung 41354, Taiwan.

**Keywords:** tripartite motif 21 (TRIM21), oral squamous cell carcinoma (OSCC), single-nucleotide polymorphism (SNP)

## Abstract

Squamous cell cancer of head and neck (HNSCC) is the sixth most common malignancy worldwide. One of the most common HNSCC types is oral squamous cell carcinoma (OSCC), which is the fifth leading cause of cancer death in Taiwan. Tripartite motif 21 (TRIM21) has been reported to play an important role in different cancer types. We found a correlation between TRIM21 and survival of HNSCC patients, but little information exists about how altered TRIM21 expression contributes to tumorigenesis. Thus, we investigated the combined effect of TRIM21 polymorphisms and exposure to environmental carcinogens on the susceptibility and clinicopathological characteristics of OSCC. Two single-nucleotide polymorphisms (SNPs) of TRIM21 (rs4144331, rs915956) from 1194 healthy controls and 1192 OSCC patients were analyzed by real-time PCR. Among 1632 smokers, TRIM21 polymorphism carriers with the betel-nut chewing habit had a ~4.8-fold greater risk of OSCC than TRIM21 wild-type carriers without the betel-nut chewing habit. After adjusting for other covariants, OSCC patients with G/T at TRIM21 rs4144331 had a high risk for distant metastasis compared with G/G homozygotes. This study is the first to examine the risk factors associated with TRIM21 SNPs in OSCC progression and development. Thus, our findings suggest that this study is the first to examine the risk factors associated with TRIM21 SNPs in OSCC progression and development and suggest that interactions between mutant genes may alter the susceptibility to OSCC.

## Introduction

Squamous cell cancer of head and neck (HNSCC) is the sixth most common malignancy worldwide, and one of the most common HNSCC types is oral squamous cell carcinoma (OSCC) 1. Despite comprehensive treatment that includes surgery, radiation, and chemotherapy, the 5-year survival rate for OSCC patients remains poor 2, 3. The poor prognosis is attributable to the development of distant metastasis and local recurrences 4, 5. OSCC occurs through a variety of genetic changes ascribed to long-term exposure to environmental carcinogens. Chronic inflammation, tobacco use, alcohol consumption, betel-nut chewing, and viral infection are all considered risk factors for OSCC 6-9. Recently, we found a correlation between Tripartite motif 21 (*TRIM21*) and HNSCC with respect to patient survival**.** TRIM proteins are composed of multidomain ubiquitin E3 ligases characterized by the presence of the N-terminal tripartite motif, which includes three zinc-binding domains - a RING, a B-box, and a coiled-coil region 10. Therefore, TRIM proteins are considered key regulators of cellular homeostasis. Recent evidence has shown that TRIM proteins can affect cell proliferation, differentiation, migration, innate immunity, and apoptosis regulation 11-15.

TRIM21 has E3 ligase activity and functions in ubiquitination. It is also known as Ro52, SSA1, or RNF81 16. In the past few years, studies of TRIM21 have been focused on its role in immunity, such as neutralizing viral infections, regulating the production of cytokine and chemokine, and regulating inflammatory signaling pathways. 17-19. TRIM21 was first identified as an antibody-binding protein. The functions of TRIM21 is as an Fc receptor that recognizes antibodies that bind to intracellular pathogens and catalyzes the synthesis of K63 ubiquitin chains resulting in activating the innate immune system and antiviral response during pathogen invasion 20, 21. Recent studies have shown that TRIM21 plays a role in the occurrence and prognosis of various tumors. Studies have reported that reduced expression of TRIM21 in hepatocellular carcinoma, breast cancer and diffuse large B-cell lymphoma implicated in a poor prognosis 22-25. In addition, TRIM21 inhibits the epithelial-mesenchymal transition (EMT) in breast cancer through the ubiquitination and degradation of Snail 24. Interestingly, it is also reported that increased expression of TRIM21 in glioma suppressed cellular senescence via the p53-p21 pathway, increased drug resistance in glioma cells and is implicated in a poor prognosis 26. However, whether TRIM21 is expressed and functions in OSCC remains unknown.

Between two randomly selected human genomes, 99.9% of the DNA sequences is individuals. The remaining 0.1% is considered to include some differences or variations in the genome between individuals. This variation is called polymorphism and is caused by mutation. Single-nucleotide polymorphisms (SNPs) are more common than other types of polymorphisms. Moreover, it occurs at a frequency of approximately 1 per 1000 base pairs in the entire genome 27. Several studies have been shown that the genomic level of humans is similar to chimpanzees. But there are differences between human and chimpanzees. For example, several diseases and cancers are common in humans but rare in chimpanzees. It is indicated that SNPs may provide important clues of cancer progression. In recent years, various researchers have reported evidence for the involvement of certain genetic predisposing factors for OSCC or HNSCC. Among genetic factors, SNPs are the most common type of DNA sequence variation and may predict cancer risk 28, 29. For instance, patients with HCC who carried at least one C allele at rs6950683 or rs3757441 have a higher risk of lymph node metastasis but a lower risk of liver cirrhosis than patients who carried the wild-type allele 24. *TRIM21* polymorphisms are also associated with autoimmune disease, and there may be a correlation between *TRIM21* polymorphisms and disease susceptibility and increased production of TRIM21 antibodies in systemic lupus erythematosus and Sjogren's syndrome. The rs660 C/T SNP has been shown to be related to systemic lupus erythematosus among African Americans 30, 31. In a Norwegian population, the rs5030767 C/T, rs5030768 A/G, rs915956 C/T, and rs4144331 C/A SNPs were shown to be associated with anti-TRIM21-positive primary Sjogren's syndrome, among which rs915956 shows the strongest association 32. However, the effects of *TRIM21* polymorphisms are still unknown in OSCC. In the present study, we aimed to investigate the association of *TRIM21* polymorphisms with OSCC. We analyzed two SNPs, rs4144331 and rs915956 for associations with demographic, etiological, and clinical characteristics and with susceptibility to OSCC.

## Materials and Methods

### Study subjects and specimen collection

This hospital-based case-control study recruited 1,192 OSCC patients as the case group between 2010 and 2019 from Sun Yat-sen Medical University Hospital in Taichung, Taiwan. The diagnosis of OSCC was performed according to the standards specified the national guidelines for OSCC. According to the tumor/lymph node metastasis criteria of the American Joint Committee on Cancer, OSCC patients were clinically staged at the time of diagnosis (2002). For the control group, all 1,194 controls were received in the same hospital, and these control individuals did not have a self-reported medical history of any cancer type. The patients' clinicopathological characteristics were verified by chart review, which including pathological staging, lymph node metastasis, and histopathologic grading levels. The whole blood samples collected from the control group and OSCC patients for TRIM21 polymorphism analysis were placed in a tube containing EDTA, centrifuged immediately, and stored at -80 °C. The research protocol has been reviewed and approved by the Taichung Zhongshan Medical University Hospital. All methods were performed in accordance with approved guidelines. Before participating in the study, all subjects provided written informed consent.

### Comprehensive Analysis of TRIM21 from the Cancer Genome Atlas (TCGA) and the Genotype-Tissue Expression (GTEx) Projects

Gene expression profile interactive analysis (GEPIA, http://gepia.cancer-pku.cn/index.html) uses standard processing pipelines to analyze RNA-Seq expression data from GTEx and TCGA, including 8,587 normal and 9,736 tumor samples in this study, we used GEPIA for tumor/normal differential expression analysis of TRIM21 expression and overall survival analysis in HNSCC.

### Selection of TRIM21 polymorphisms

For the present study, we selected two SNPs in TRIM21 (NM_003141.4) from the International HapMap Project data. We included the SNPs rs4144331 and rs915956, which are respectively located in intron 3 (9571) and the 3'-untranslated regions (12986) of TRIM21.

### TRIM21 Genotyping

The allelic discrimination of TRIM21 polymorphisms rs4144331 and rs915956 was evaluated using the ABI StepOne Real-Time PCR system (Applied Biosystems), the TaqMan assay, and SDS v3.0 software (Applied Biosystems) 17-19. The final volume for each reaction was 5 μL, containing 0.125 μL TaqMan probes mix, 2.5 μL TaqMan Genotyping Master Mix, and 10 ng genomic DNA. The reaction conditions included an initial denaturation step at 95 °C for 10 minutes, followed by 40 cycles at 95 °C for 15 seconds and 60 °C for 1 minute.

### Statistical analysis

The differences in age and demographic characteristics between control and OSCC patients was compared by the Mann-Whitney U-test. The odds ratios (ORs) with 95% confidence intervals (CIs) were estimated by logistic regression models. After controlling for other covariates, the adjusted odds ratios (AORs) with 95% CIs of the association which are between genotype frequencies and OSCC risk as well as clinicopathological characteristics were estimated by multiple logistic regression models. Values of p < 0.05 were considered significant. All the data were analyzed using SAS statistical software (Version 9.1, 2005; SAS Institute Inc., Cary, NC).

## Results

**Figure 1:**
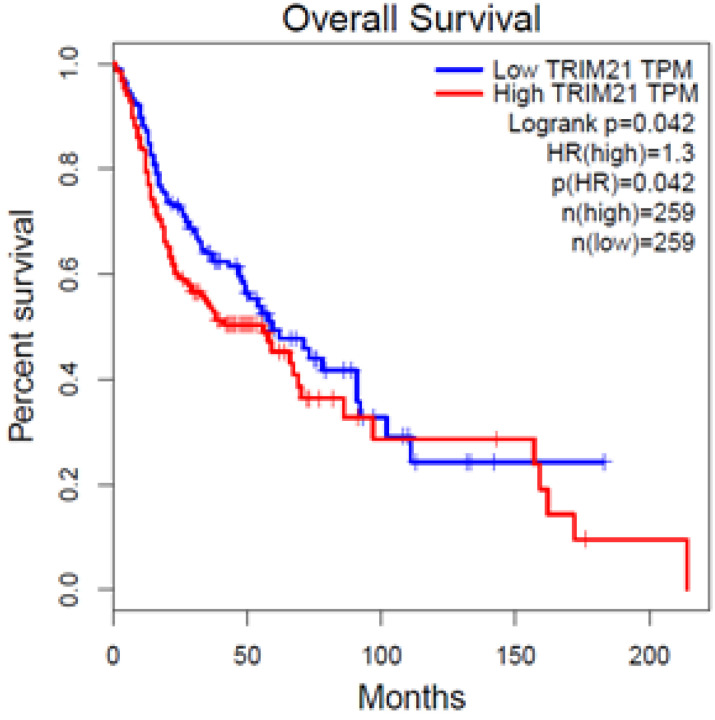
Upregulated expression of *TRIM21* is associated with poor prognosis of patients with squamous cell cancer of head and neck (HNSCC), as assessed with data from the Gene Expression Profiling Interactive Analysis (GEPIA).

To investigate the clinical impact of *TRIM21* on HNSCC cancer progression, we used GEPIA to assess the relationship between cellular levels of* TRIM21* mRNA and HNSCC patient outcomes. The HNSCC patients with high *TRIM21* expression had significantly shorter overall survival than those with low *TRIM21* expression (Figure 1). This result implies that enhanced expression of *TRIM21* might be involved in HNSCC progression.

We recruited 1,194 healthy controls and 1,192 patients with OSCC for this case-cohort study. The demographic characteristics and the etiological and clinical characteristics of OSCC patients revealed that the mean age did not differ significantly between people with (n =1,192) or without OSCC (n = 1,194) (Table 1). There were significant differences between groups of betel nut chewing (*p* <0.001), smoking (*p* <0.001), and drinking (*p* <0.001). All behaviors are more common in the OSCC cohort than in the control group (Table 1). Approximately half of the patients (47.3%) had stage I/II cancer, and half (52.7%) had stage III/IV cancer (Table 1). One-third (32.7%) had N1-N3 lymph node metastasis. Approximately all tumors (99.2%) were classified as M0 status, and the majority of tumors (85.7%) were moderately or poorly differentiated (Table 1).

To reduce the possible interference from several environmental factors, the AORs and their corresponding 95% CIs were estimated after controlling for the risk related to age, alcohol consumption, and tobacco use for each comparison by multiple logistic regression models. The distribution frequency of *TRIM21* genotypes for controls and OSCC patients is shown in Table 2. For the controls, all genotypic frequencies were in Hardy-Weinberg equilibrium (*p* > 0.05). For both patients and controls, most of those with the rs4144331 and rs915956 SNPs were homozygous for the G/G genotype. There was no significant difference with respect to the *TRIM21* rs4144331 and rs915956 polymorphisms between controls and patients with OSCC.

According to recent research, tobacco use and betel-nut chewing are important risk factors for OSCC progression 6-8. Genotyping and allele frequency data for *TRIM21* SNPs among smokers are shown in Table 3. Among all 1,632 smokers, those who either had at least one T allele of rs4144331, one A allele of rs915956, and chewed betel nuts were 9.541-fold (95% CI: 6.784-13.418) and 10.131-fold (95% CI: 7.066-14.525), respectively, more likely to have OSCC than wild-type homozygous smokers who did not chew betel nuts. Furthermore, smokers with at least one T allele of rs4144331, one A allele of rs915956, and who chewed betel nuts had respective risks that were 4.834-fold (95% CI: 3.691-6.333) and 4.804-fold (95% CI: 3.717-6.209) higher, respectively, than the wild-type homozygous smokers for developing OSCC. These results indicated that *TRIM21* polymorphisms have a great influence and significant difference on oral cancer susceptibility in men who smoke tobacco and/or chew betel nuts.

The AORs with their corresponding 95% CIs were estimated by multiple logistic regression models after controlling for age and alcohol consumption.

We further compared associations between the *TRIM21* rs4144331 polymorphism and clinical status of OSCC patients (Table 4). Compared with patients with the G/G genotype, those with the G/T genotype at the rs4144331 SNP were 4.294-fold (95% CI: 1.104-16.696) more likely to have metastasis (*p* = 0.036). No significant between-group differences were observed for pathological stage, tumor size, lymph node metastasis, or cell differentiation at the rs4144331 SNP.

## Discussion

SNPs are nucleotide variations that occur at the DNA level in every human cell. Similar to the potential impacts of environmental factors, SNPs can mimic human phenotypic diversity, but they also can be correlated with susceptibility to a variety of diseases including cancer 28. To distinguish the effects of SNPs from those of other types of gene mutations, the incidence of each polymorphism must be greater than the rate of a single natural mutation. Changes in the function of gene products due to mutations or genetic polymorphisms may contribute to increased cancer risk and certain disease phenotypes 29.

We mentioned that TRIM21 might play an important role in the occurrence and prognosis of various tumors. Over expression of TRIM21 in glioma suppressed cellular senescence via the p53-p21 pathway, increased drug resistance in glioma cells and is implicated in a poor prognosis 26. Therefore, in this study, we evaluated the expression of *TRIM21* in head and neck cancer based on TCGA database. The result indicated that the HNSCC patients with high *TRIM21* expression had significantly shorter overall survival than those with low *TRIM21* expression (Figure 1). Based on the above, we can infer that TRIM21 as a potential marker for prognosis in HNSCC or OSCC. Although the relationship between TRIM21 alleles and disease susceptibility has been extensively studied, the correlation between TRIM21 polymorphisms and environmental risk factors for OSCC has not been clarified. The frequency of rs915956 (C/C) in anti-TRIM21-positive patients with Sjogren's syndrome is about one-half that in healthy controls 30. Therefore, our results provide novel information about how TRIM21 SNPs might contribute to OSCC susceptibility, the interaction of those SNPs with environmental risk factors, and clinicopathological conditions.

Exposure to environmental carcinogens may promote oral cancer. Genomic changes during OSCC will gradually change the OSCC phenotype, resulting in cell intermediates that could potentially evolve into malignant tumors 31. Polymorphisms of several genes are associated with increased the risk of OSCC 32. Thus, genetic components may play a pivotal role in carcinogenesis. In our hospital-based case-control study, our results revealed significant between-group differences for betel-nut chewing, cigarette smoking, and alcohol consumption; all behaviors were significantly more prevalent among the OSCC cohort compared with controls (Table 1). This result indicated environmental factors are highly associated to the increased risk of OCSS. Exposure to environmental carcinogens may be involved in the process of tumorigenesis. However, there are more evidence suggests that genetic variants may be more helpful in predicting cancer risk 33, 34. Therefore, the relationship between SNPs and OSCC risk is analyzed by controlling the environmental impact.

The distribution frequency of TRIM21 genotypes for controls and OSCC patients is shown in Table 2. However, there was no significant difference with respect to the TRIM21 rs4144331 and rs915956 polymorphisms between controls and patients with OSCC. Even through the well-known is that environmental factors and chemicals might elicit epigenetic changes which increased the rates of caners and other diseases. These two TRIM21 SNPs were shown to be associated with anti-TRIM21-positive primary Sjogren's syndrome 32. Recent studies have shown that cigarette smoke induced DNA damage and cancer associated epigenomic alterations in lung cancer 35, 36. Moreover, it has been detected that the absence of *CDKN2A*,* p14*,* p15*, and *p16* promoter hypermethylation in tumors from betel-quid chewing patients 37, 38. In this study, we have found that TRIM21 polymorphisms have a strong impact and significant difference on oral cancer susceptibility in men who smoke tobacco and/or chew betel nuts (Table 3). The reason why these two polymorphisms did not act as driver gene in OSCC remains unclear and needs further evaluation. Nevertheless, TRIM21 SNPs can promote OSCC progression while the cancer is occurred. Therefore, we further compared associations between the TRIM21 rs4144331 polymorphism and clinical status of OSCC patients (Table 4). Compared with patients with the G/G genotype for the rs4144331 SNP, those with the G/T genotype were 4.294-fold more likely to progress to a metastatic state. Although the sample size is rare in M1. In fact, it is hard to collect the M1 patient in clinical. Thus, we will need to collect these M1 patient to confirm the detail relationship in the future. Finally, considering the association between TRIM21 rs4144331 SNP and other common somatic genetic changes in OSCC, the constitutional rs4144331 SNP may be an important determinant of predicting tumor recurrence after treatment, response to targeted therapy, and drug toxicity.

## Conclusions

Our findings suggest that the interaction of clinical features of genes may alter the susceptibility to OSCC. This study provides new information about the relationship between TRIM21 polymorphisms and OSCC clinical pathology in the Taiwanese population.

## Figures and Tables

**Table 1:** Demographic characteristics of controls and OSCC patients

Variable	Controls (n=1194)	Patients (n=1192)	*p* value
**Age (years)**			0.969
<55	563 (47.1%)	563 (47.2%)	
≥55	631 (52.9%)	629 (52.8%)	
**Betel-nut chewing**			< 0.001*
No	996 (83.4%)	322 (27.0%)	
Yes	198 (16.6%)	870 (73.0%)	
**Cigarette smoking**			< 0.001*
No	561 (47.0%)	193 (16.2%)	
Yes	633 (53.0%)	999 (83.8%)	
**Alcohol consumption**			< 0.001*
No	958 (80.2%)	647 (54.3%)	
Yes	236 (19.8%)	545 (45.7%)	
**Stage**			
I+II		564 (47.3%)	
III+IV		628 (52.7%)	
**Tumor T status**			
T1+T2		599 (50.3%)	
T3+T4		593 (49.7%)	
**Lymph node status**			
N0		802 (67.3%)	
N1+N2+N3		390 (32.7%)	
**Metastasis**			
M0		1182 (99.2%)	
M1		10 (0.8%)	
**Cell differentiation**			
Well differentiated		171 (14.3%)	
Moderately or poorly differentiated		1021 (85.7%)	

Mann-Whitney U test or Chi-square test was used to assess the significance of differences between healthy controls and patients with OSCC. *p < 0.05 as statistically significant.

**Table 2:** Genotyping and allele frequency of *TRIM-21* SNPs among controls and OSCC patients

Variable	Controls (total n=1194) n (%)	Patients (total n=1192) n (%)	OR (95% CI)	AOR (95% CI)^a^
**rs4144331**				
GG	720 (60.3%)	728 (61.1%)	1.000 (reference)	1.000 (reference)
GT	423 (35.4%)	401 (33.6%)	0.938 (0.790-1.113)	0.928 (0.752-1.145)
TT	51 (4.3%)	63 (5.3%)	1.222 (0.833-1.792)	1.461 (0.915-2.333)
GT+TT	474 (39.7%)	464 (38.9%)	0.968 (0.821-1.141)	0.980 (0.801-1.199)
**rs915956**				
GG	830 (69.5%)	818 (68.6%)	1.000 (reference)	1.000 (reference)
GA	320 (26.8%)	344 (28.9%)	1.091 (0.911-1.306)	0.967 (0.775-1.207)
AA	44 (3.7%)	30 (2.5%)	0.692 (0.431-1.111)	0.750 (0.416-1.351)
GA+AA	364 (30.5%)	374 (31.4%)	1.043 (0.876-1.240)	0.944 (0.762-1.169)

The ORs, along with their corresponding 95% Cis, were estimated by logistic regression models. ^a^Adjusted for the effects of age, betel quid chewing, cigarette smoking, and alcohol consumption.

**Table 3:** Associations of the combined effect of *TRIM-21* polymorphisms and betel nut chewing on susceptibility to oral cancer among 1632 smokers

Variable	Controls (n=633) (%)	Patients (n=999) (%)	OR (95% CI)	AOR (95% CI)
**rs4144331**				
^a^GG genotype & non-betel-nut chewing	277 (43.8%)	107 (10.7%)	1.00 (reference)	1.000 (reference)
^b^GT or TT genotype or betel-nut chewing	280 (44.2%)	575 (57.6%)	**5.316 (4.079-7.519) *p*<0.001**	**4.834 (3.691-6.333) *p* <0.001**
^c^GT or TT genotype with betel-nut chewing	76 (12.0%)	317 (31.7%)	**10.798(7.722-15.099)*p* <0.001**	**9.541(6.784-13.418)*p* <0.001**
				
**rs915956**				
^a^GG genotype & non-betel-nut chewing	303 (47.9%)	126 (12.6%)	1.00 (reference)	1.000 (reference)
^b^GA or AA genotype or betel-nut chewing	274 (43.3%)	601 (60.2%)	**5.275 (4.099-6.787) *p* <0.001**	**4.804 (3.717-6.209) *p* <0.001**
^c^GA or AA genotype with betel-nut chewing	56 (8.8%)	272 (27.2%)	**11.680(8.191-16.654) *p* <0.001**	**10.131(7.066-14.525) *p* <0.001**

**Table 4:** OR and 95% CI values of clinical status categories associated with genotypic frequencies of *TRIM-21* rs4144331 in male oral cancer patients (n = 1192)

Variable			OR (95% CI)	*p* value
	**Pathological Stage**			
**rs4144331**	Stage I+II (n=564) (%)	Stage III+IV (n=628) (%)		
GG	351 (62.2%)	377 (60.0%)	1.00	
GT	183 (32.4%)	218 (34.7%)	1.109 (0.869-1.416)	0.406
TT	30 (5.3%)	33 (5.3%)	1.024 (0.612-1.715)	0.928
	**Tumor size**			
**rs4144331**	≤ T2 (n=599) (%)	> T2 (n=593) (%)		
GG	373 (62.3%)	355 (59.9%)	1.00	
GT	198 (33.0%)	203 (34.2%)	1.077 (0.844-1.375)	0.550
TT	28 (4.7%)	35 (5.9%)	1.313 (0.783-2.204)	0.301
	**Lymph node metastasis**			
**rs4144331**	No (n=802) (%)	Yes (n=390) (%)		
GG	492 (61.3%)	236 (60.5%)	1.00	
GT	265 (33.0%)	136 (34.9%)	1.070 (0.826-1.386)	0.608
TT	45 (5.7%)	18 (4.6%)	0.834 (0.472-1.472)	0.530
	**Metastasis**			
**rs4144331**	M0 (n=1182) (%)	M1 (n=10) (%)		
GG	725 (61.3%)	3 (30.0%)	1.00	
GT	394 (33.3%)	7 (70.0%)	4.294 (1.104-16.696)	0.036^*^
TT	63 (5.3%)	0 (0.0%)	-	-
	**Cell differentiation grade**			
**rs4144331**	≤ Grade I (n=171) (%)	> Grade I (n=1021) (%)		
GG	107 (62.6%)	621 (60.8%)	1.00	
GT	52 (30.4)	349 (34.2%)	1.156 (0.810-1.651)	0.424
TT	12 (7.0%)	51 (5.0%)	0.732 (0.378-1.418)	0.355

Cell differentiation grade: grade I, well differentiated; grade II, moderately differentiated; grade III, poorly differentiated.
